# The Forehead Flap

**Published:** 2014-12-30

**Authors:** Adrian Frunza, Amit Beedasy, Adriana Anghel, Ioan Lascar

**Affiliations:** Bucharest Emergency Clinical Hospital, Bucharest University School of Medicine, Romania

**Keywords:** cheek reconstruction, forehead flap, cheek defects, forehead blood supply, forehead esthetic unit

## DESCRIPTION

A 84-year-old woman came in our clinic with a exophytic, ulcerated, hemorrhagic tumor of the right cheek. The computed tomographic scan showed tumoral involvement of the facial muscles with extension to the anterior maxillary sinus wall. The pathological examination found a moderately differentiated squamous cell carcinoma.

## QUESTIONS

1. What are the key principles of cheek reconstruction?
2. What are the anatomical features?
3. Which are the clinical variants?
4. What are the indications, and how the injuries are managed by the use of the forehead flap?

## DISCUSSION

The cheeks represent the largest surface area of the face and frame the central facial units. Reconstruction must be planned carefully to restore the natural contours, maintain hair patterns, and camouflage scars. For any given defect, more than 1 reconstructive option is usually available. Reconstruction of defects involving the full thickness of the cheek implies reconstruction of all layers, while maintaining reasonable contour.^1^^,^^2^

The forehead flap is acknowledged as the ideal donor for midface reconstruction due to its color and texture match, vascularity, and ability to resurface all or part of the reconstructed area.^3^ The flap is usually designed as an esthetic unit and comprises the hairless skin of the forehead between the scalp and the eyebrows and extends laterally to the preauricular and temporal hair-bearing skin. The inferior border of the flap should lie immediately above the eyebrow, primarily to include the inferior branch of the superficial temporal artery but also to improve the donor site deformity by the use of a skin graft as a forehead esthetic unit. The flap is generally elevated at the level of the periosteum. It is possible to elevate the flap superficially to the frontalis and corrugator muscles to preserve facial expression.^4^ The superficial temporal artery is considered the dominant pedicle of the flap. The motor inervation of the flap is through the frontal branch of cranial nerve VII (of the facial nerve). Brow elevation function is completely lost with the use of the standard forehead flap. The sensory nerves supplying the flap arise from supratrochlear, supraorbital, and auriculotemporal nerves.^3^^,^^4^^,^^5^

There are 4 clinical variants. The standard forehead flap (temporal based-flap) includes all or part of the forehead skin. Pertinent landmarks are the anterior hairline and superior edge of the eyebrow. The modified forehead flap places the markings more posteriorly in the bald patient so that the skin over the frontal skull can be used. A small island flap may also be designed. The posterior bicoronal incision represents the posterior aspect of the extended scalp flap. The skin island of the reverse flap is designed in the preauricular skin between the zygomatic arch and ear lobule.^4^ The bilobed forehead flap using both branches of the superficial temporal artery can be used to cover both the mucosal and skin surfaces in full-thickness cheek defects; the forehead flap can be used to reconstruct the intraoral mucosa and the scalp flap can be used for external skin coverage.^6^

The standard flap extends to the middle and lower third of the face and the oral cavity. The extended scalp flap is designed to reach the middle third of the face (for nasal reconstruction). The island flap will compensate midface defects and oral cavity defects, passed through a cheek tunnel. The reverse flow island flap will reach defects on the ispsilateral forehead and orbit.^4^

The forehead flap will cover the ipsilateral cheek, eye, and nasal defects, and therefore it is used after tumor resection, congenital anomalies surgery, trauma, and degenerative diseases; a forehead flap is long enough to provide cover to the carotid artery in the upper neck. It also can be used to reconstruct the cervical esophagus, provided that it is passed under the malar region and the body of the mandible is removed.^6^^,^^7^ Despite its versatility, nowadays it is rarely used because of the drawbacks involving the donor site coverage (grafted periosteum). One of the alternatives to improve the donor site aspect is to use Integra or any dermal substitutes. These were not available in our case.

## Figures and Tables

**Figure 1:**
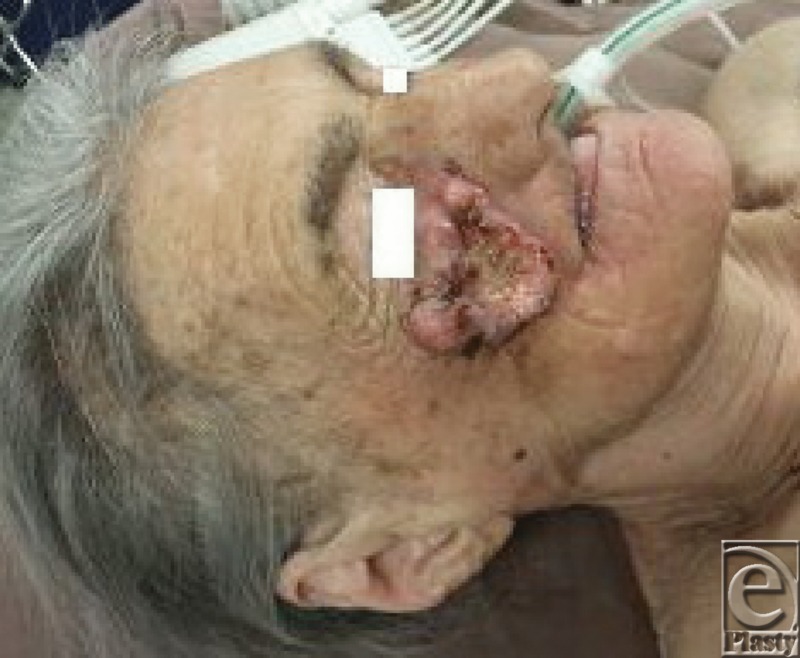
Preoperative aspect.

**Figure 2:**
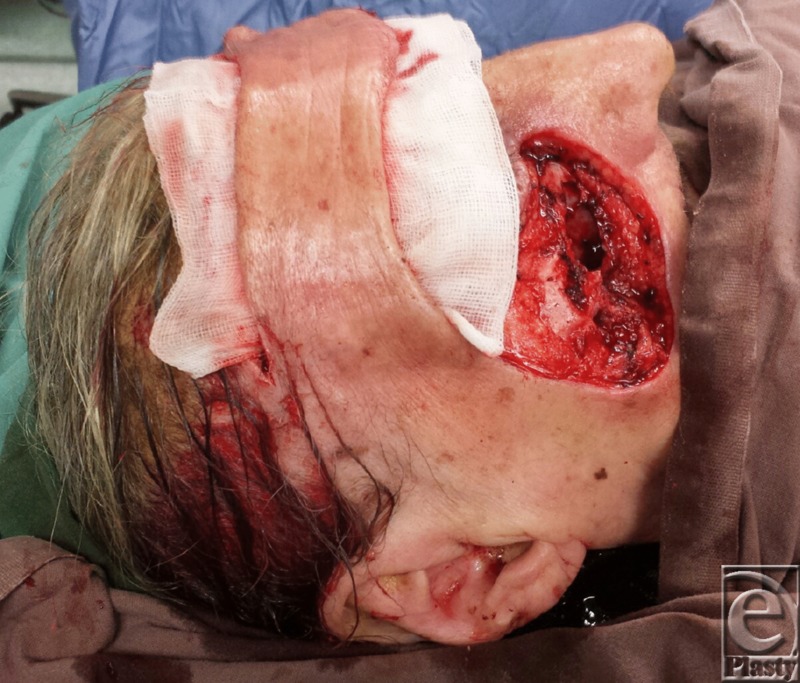
Forehead flap raised with right lateral pedicle and the defect to be covered.

**Figure 3:**
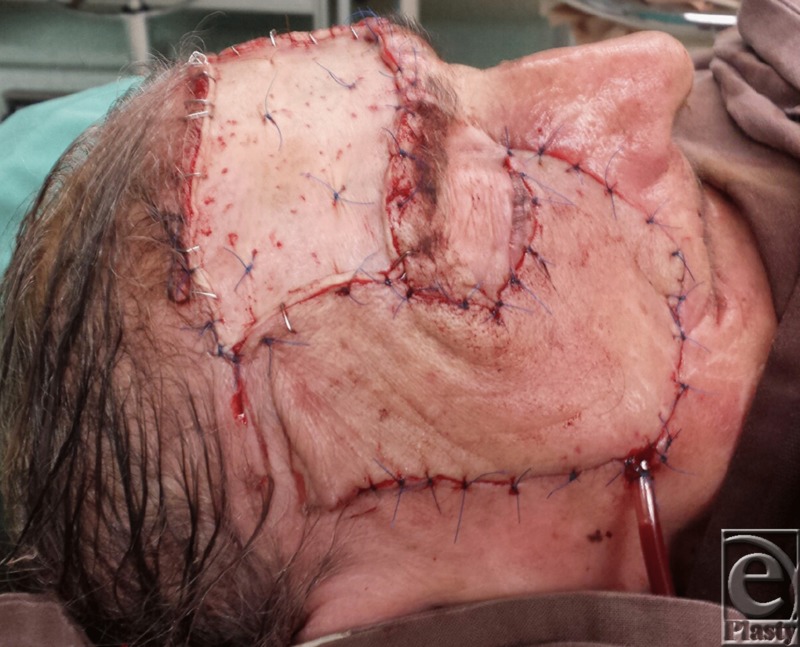
Immediate postoperative aspect: the forehead flap covering the cheek defect; the secondary defect covered by split-thickness skin graft.

**Figure 4:**
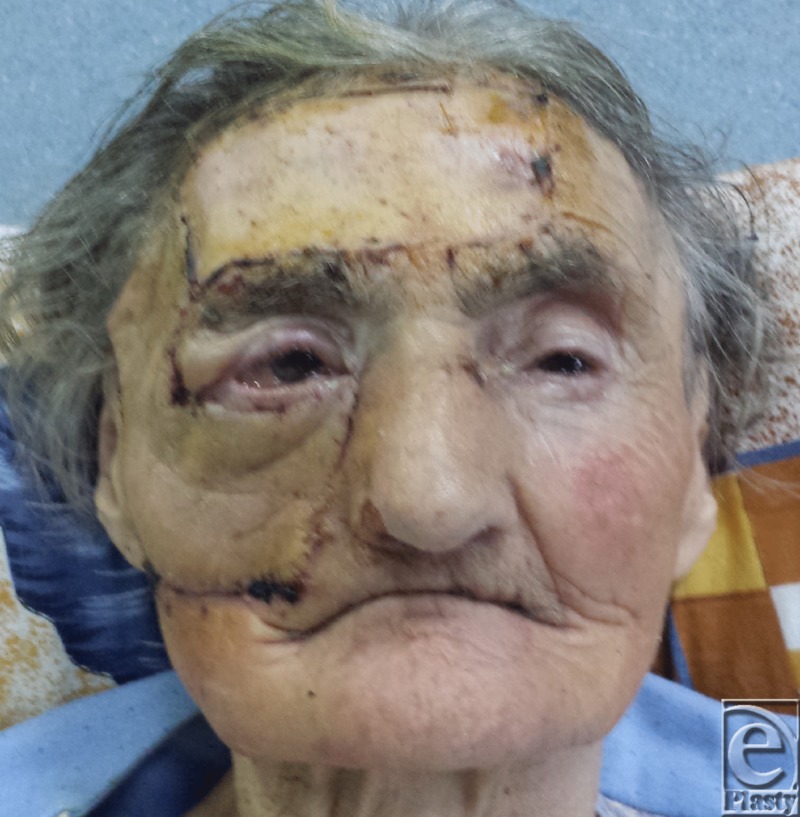
Postoperative aspect at 10 days.
